# Range contraction and increasing isolation of a polar bear subpopulation in an era of sea‐ice loss

**DOI:** 10.1002/ece3.3809

**Published:** 2018-01-18

**Authors:** Kristin L. Laidre, Erik W. Born, Stephen N. Atkinson, Øystein Wiig, Liselotte W. Andersen, Nicholas J. Lunn, Markus Dyck, Eric V. Regehr, Richard McGovern, Patrick Heagerty

**Affiliations:** ^1^ Polar Science Center Applied Physics Laboratory University of Washington Seattle WA USA; ^2^ Greenland Institute of Natural Resources Nuuk Greenland; ^3^ Wildlife Research Section Department of Environment Government of Nunavut Igloolik NU Canada; ^4^ Natural History Museum University of Oslo Oslo Norway; ^5^ Department of Bioscience Aarhus University Rønde Denmark; ^6^ Environment and Climate Change Canada University of Alberta Edmonton AB Canada; ^7^ Department of Biostatistics University of Washington Seattle WA USA

**Keywords:** animal movements, Arctic, contraction, isolation, polar bear, range, sea ice

## Abstract

Climate change is expected to result in range shifts and habitat fragmentation for many species. In the Arctic, loss of sea ice will reduce barriers to dispersal or eliminate movement corridors, resulting in increased connectivity or geographic isolation with sweeping implications for conservation. We used satellite telemetry, data from individually marked animals (research and harvest), and microsatellite genetic data to examine changes in geographic range, emigration, and interpopulation connectivity of the Baffin Bay (BB) polar bear (*Ursus maritimus*) subpopulation over a 25‐year period of sea‐ice loss. Satellite telemetry collected from *n* = 43 (1991–1995) and 38 (2009–2015) adult females revealed a significant contraction in subpopulation range size (95% bivariate normal kernel range) in most months and seasons, with the most marked reduction being a 70% decline in summer from 716,000 km^2^ (SE 58,000) to 211,000 km^2^ (SE 23,000) (*p* < .001). Between the 1990s and 2000s, there was a significant shift northward during the on‐ice seasons (2.6^°^ shift in winter median latitude, 1.1^°^ shift in spring median latitude) and a significant range contraction in the ice‐free summers. Bears in the 2000s were less likely to leave BB, with significant reductions in the numbers of bears moving into Davis Strait (DS) in winter and Lancaster Sound (LS) in summer. Harvest recoveries suggested both short and long‐term fidelity to BB remained high over both periods (83–99% of marked bears remained in BB). Genetic analyses using eight polymorphic microsatellites confirmed a previously documented differentiation between BB, DS, and LS; yet weakly differentiated BB from Kane Basin (KB) for the first time. Our results provide the first multiple lines of evidence for an increasingly geographically and functionally isolated subpopulation of polar bears in the context of long‐term sea‐ice loss. This may be indicative of future patterns for other polar bear subpopulations under climate change.

## 1 INTRODUCTION

Conservation of wildlife populations across dynamic landscapes requires information on population structure and boundaries for establishing a valid ecological and demographic basis for determining population abundance, rates of population change, and the impacts of human activities (Thomas, 2010; Weinbaum, Brashares, Golden, & Getz, 2013). In an era of climate change, barriers to dispersal are quickly being altered, which can facilitate functional or genetic transfer between animals where it was once absent, or lead to increasing isolation because corridors of exchange are no longer available (Ching Chen, Hill, Ohlemuller, Roy, & Thomas, 2011; Rubidge et al., 2012). Concurrently, many species are shifting to higher latitudes and higher elevations in response to a warming earth (Loarie et al., 2009). Shifts or reductions in ranges will likely result in reduced genetic diversity and in extreme cases, genetic impoverishment that may reduce population viability (Yannic et al., 2013). This can have rapid and sweeping impacts on conservation of large mobile species, especially those subject to subsistence harvests (Laidre et al., 2015).

The Arctic is losing sea ice at a rapid rate (Overland & Wang, 2013). This large‐scale habitat change is predicted to have multiple and significant consequences for ice‐dependent species (Laidre et al., 2015). A key question is whether loss of sea ice will reduce or enhance dispersal, given sea ice serves as both a platform for movement and a physical barrier. In the case of cetaceans, loss of sea ice has opened new habitats and resulted in positive population responses (George, Druckenmiller, Laidre, Suydam, & Person, 2015); however, this has generally not been the case for other species that rely on sea ice for key aspects of their life history, such as polar bears (*Ursus maritimus*; Amstrup & Gardner, 1994; PBSG 2010; Regehr et al., 2016). Contrasting patterns may emerge depending on a species’ dispersal ability and how changes in sea‐ice extent, formation, or break‐up structure habitat (e.g., Travis et al., 2013).

Polar bears are found throughout the circumpolar Arctic in 19 subpopulations that differ in productivity, size, and degree of connectedness with adjacent subpopulations (PBSG 2010). In areas with multiyear sea ice, some polar bears may remain on sea ice year‐round, however, in areas where the annual sea ice melts completely in summer most bears spend up to several months on land, largely fasting until freeze‐up (e.g., Stirling, Lunn, & Iacozza, 1999; Stirling & Parkinson, 2006).

**Figure 1:**
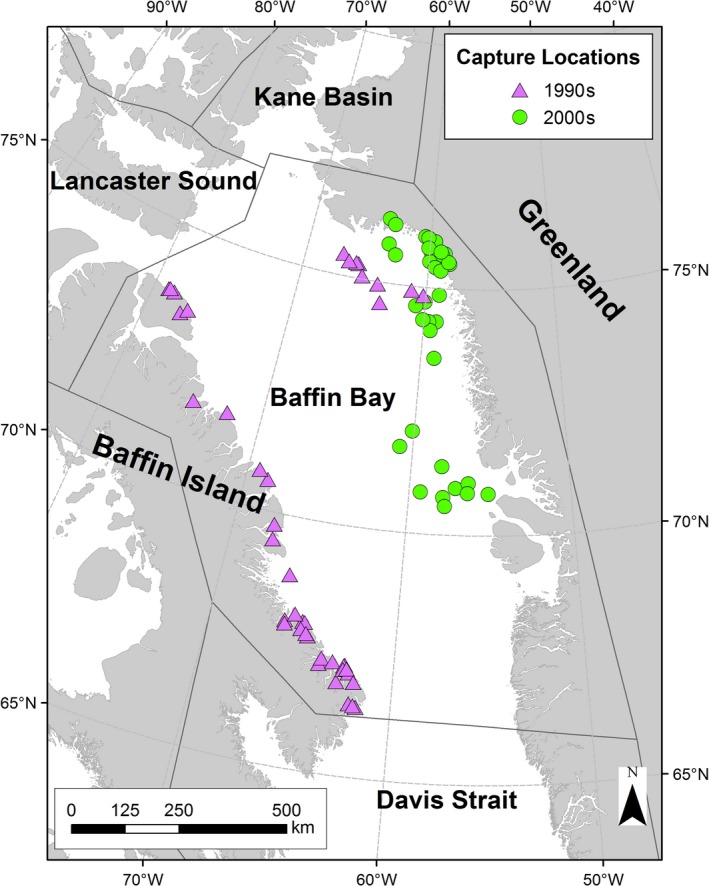
Distribution of capture locations of adult female polar bears fitted with satellite collars in Baffin Bay during the 1990s and 2000s

The Baffin Bay (BB) subpopulation of polar bears is located in the seasonal ice ecoregion where sea ice forms and disappears each year (Amstrup, Marcot, & Douglas, 2008; Taylor et al., 2001; Figure 1). In the 1990s, analyses of movements from satellite‐collared bears (Taylor et al., 2001), genetic analyses (Paetkau et al., 1999), and recaptures and harvest recoveries of individually marked bears (Taylor & Lee, 1995; Taylor et al., 2001) indicated limited interchange among BB and adjacent subpopulations including Davis Strait (DS) and Lancaster Sound (LS; Taylor et al., 2001). However, BB bears could not be genetically differentiated from the adjacent Kane Basin (KB) subpopulation to the north (Paetkau et al., 1999). As those studies, the BB subpopulation has been considered a distinct demographic unit with its dynamics largely driven by intrinsic rates of reproduction and mortality rather than exchange between neighboring subpopulations (SWG 2016). The BB subpopulation currently supports a subsistence harvest of approximately 134 bears per year, which provides a nutritional, cultural, and economic resource to Inuit communities (PBSG 2010). Declining sea ice in Baffin Bay has resulted in a lengthening of the ice‐free season by >12 days per decade during the period 1979–2014 (Stern & Laidre, 2016). There is evidence for positive correlation between sea‐ice availability and BB subpopulation reproductive rates (SWG, 2016). Furthermore, recent studies suggest low apparent survival for male polar bears in BB, which may reflect either increased natural mortality or emigration (SWG, 2016).

We conducted an assessment of the extent to which sea ice trends have affected the distribution and boundaries of the BB subpopulation and discuss results in the context of other polar bear subpopulations facing sea‐ice loss, as well as near‐term management and long‐term conservation planning. We used home range estimators from satellite telemetry to delineate subpopulation range boundaries, movement data from collared adult females and from the recapture or harvest of marked individuals to assess latitudinal shifts or changes in immigration/emigration, and genetic analyses to assess relationships between BB and adjacent subpopulations. Our objective was to explore multiple lines of evidence with respect to the delineation of the BB subpopulation in an era of rapid sea‐ice loss. We discuss the implications of the results for effective conservation and management strategies and identify where additional research and monitoring will be needed in the future.

## 2 MATERIALS AND METHODS

### 2.1 Study area

The boundaries of the BB subpopulation encompass ~1 million km^2^, covering approximately 656,000 km^2^ of ocean as well as portions of Baffin Island, all of Bylot Island in Nunavut/Canada, and parts of West and Northwest Greenland (66**°**N to 77**°**N; Taylor et al., 2005) (Figure 1). During late spring and summer break‐up, sea ice recedes from Greenland westward across Baffin Bay; the last remnants of ice typically occur off the coast of Baffin Island. Most polar bears remain on the sea ice as it recedes and then come ashore to spend the ice‐free period on Baffin and Bylot Islands (Taylor et al., 2005). An unknown but probably small number of bears remain on land in Melville Bay, NW Greenland throughout the ice‐free period (Born, 1995; Born, Heilmann, Holm, & Laidre, 2011; SWG, 2016).

### 2.2 Radio telemetry

#### 2.2.1 Handling and collaring

Adult female polar bears (AF) were captured in NW Greenland on the fast and pack ice between mid‐March and mid‐April 2009–2013 in Baffin Bay. Searches for bears occurred between 70° 22′ N and 76° 20′ N, to a maximum distance of ca. 150 km from the coast and included areas at glacier fronts. Bears were located and captured from a helicopter using standard chemical immobilization techniques (Stirling, Spencer, & Andriashek, 1989). Field estimates of age and reproductive status were recorded, with age later estimated by counting cementum growth layers on a premolar tooth extracted during capture (Calvert & Ramsay, 1998). Thirty‐eight AF were fitted with Argos‐linked satellite transmitters (i.e., Platform Transmitter Terminal, or PTT) (Model TAW‐4610H, Telonics, Mesa, Arizona, USA) (Table 1, Figure 1), which provided information on geographic location, internal transmitter temperature, and activity. These data were combined with a historical data set of 43 AF that were collared within the BB subpopulation management boundaries (PBSG, 2010) between 1991 and 1995 (Ferguson, Taylor, & Messier, 1997; Taylor et al., 2001). The majority of the 1990s tags were deployed during the ice‐free season in fall on Baffin Island (with the exception of *n* = 9 deployed in spring in NW Greenland).

**Table 1:** Number of adult female (AF) polar bears collared in Baffin Bay in the 1990s and 2000s in relation to accompanying bears (AM, adult male in breeding pair; COY, dependent cub‐of‐the‐year; YRLG, dependent yearling cub; 2YR, dependent 2‐year‐old cub)

	AF alone	AF+AM	AF+COY	AF+YRLG	AF+2YR	Sum
1990s	9	0	19	13	2	43
2000s	10	2	6	12	8	38

#### 2.2.2 Data filtering and sub‐sampling

Locations and transmitter status were collected via the Argos Location Service Plus system (Toulouse, France). The quality of each location was assigned by ARGOS with location qualities of 0–3 estimated to have errors of 1.5 km or less and those categorized as “A,” “B,” or “Z” had no predicted accuracy. Unrealistic and poor quality locations were removed using a speed and angle filter in R version 2.13.2 (R Development Core Team 2012) using the package “argosfilter” (Freitas, Kovacs, Lydersen, & Ims, 2008). Positions exceeding a maximum between‐location travel velocity (10 km/hr based on previous movement studies of polar bears, Laidre et al., 2013) and angle (measured from the track between three successive locations; set to the default) were removed by the filtering algorithm. The resulting locations for each bear were reduced to a single position per day to reduce autocorrelation bias, standardize temporal sampling, and address the effects of variable duty cycling among PTTs. The first, best quality location within the period of peak satellite passage was selected to obtain a daily position for each PTT. Daily positions, after filtering and optimal daily position selection, only consisted of ARGOS qualities 1–3. Distances between successive daily positions were calculated as the great circle route and used to compute minimum daily displacements.

As a result of variable experimental objectives in both decades, different duty cycles were used for tags in an effort to extend battery life or gather information from specific time periods. Collars in the 2000s were programmed to transmit during one 6‐hr period each day on 4‐day intervals. The 1990s collars were programmed to transmit on varying and intermittent intervals, ranging from 1 to 6 days. We subsampled the 1990s data and created a strict 4‐, 5‐, or 6‐day interval time series for each individual to best match the 2000s data. This ensured that serial autocorrelation was consistent among decades.

Telemetry data were divided into seasons: spring (March–July, which included the peak of sea‐ice coverage and initiation of sea‐ice break‐up), summer (August–October, which included the end of break‐up and the on‐land period), and winter (November–February, which included the freeze‐up period and time when bears returned to the sea ice). All periods when collared females were denning (use of maternity and shelter dens) were identified (Escajeda et al., 2018) and removed from analyses. Bears with <3 locations were removed from analyses when transmitter failure occurred immediately after deployment.

#### 2.2.3 Comparison of 1990s and 2000s BB satellite telemetry data

Polar bears collared in this study ranged over the entire Baffin Bay region (SWG, 2016). All captures occurred within the bounds of the BB subpopulation management unit (PBSG, 2010) and bears moved back and forth freely between Canada and Greenland during both periods (Figure 1). However, seasonal and geographic differences in capture locations occurred in our study between the two periods of fieldwork. In the 1990s, it was possible to deploy collars on AFs on both the Canadian and Greenlandic sides of Baffin Bay (Figure 1). However, in the 2000s, it was only possible to deploy collars on the Greenlandic side of Baffin Bay due to community reservations in Nunavut about polar bear immobilization and handling (SWG, 2016). This reservation was not shared in Greenland by hunters and management authorities.

Prior to conducting interdecadal analyses of polar bear movement data, we evaluated the 1990s and 2000s data to identify potential effects of differences in collar deployment location. First, we compared the fall telemetry locations of bears collared on the Greenlandic side in spring in the 2000s, with the dates of the fall capture locations of bears collared on the Canadian side (on Baffin Island) in the 1990s. We then quantified what overall range of latitudes on land on Baffin Island was used by polar bears in both decades. Finally, we subset the 1990s dataset to conduct interdecadal analyses on range size using a subsample of 1990s bears (*n* = 9) collared in spring on the sea ice in Greenland compared with range size of bears collared in the same area in the 2000s (see Figure 1). This essentially replicated some analyses we present in this manuscript (but with a substantially reduced sample size in the 1990s).

Overall, excluding permanent residents of Melville Bay, 92% of the AF bears collared in spring in West Greenland in the 2000s moved to Baffin Island by fall and were located inside the same fall collaring area used in the 1990s (defined as a polygon around 1990s collaring sites). Further, AF bears collared in West Greenland in the 2000s were distributed over the same range of on‐land latitudes on Baffin Island in fall as those collared in the 1990s (66^°^30′ N to 73^°^50′ N), confirming bears from West Greenland use the same areas as those captured in the 1990s. Finally, our analyses using the subset of 1990s AF bears collared in spring on the sea ice in West Greenland (*n* = 9) compared to the full sample of 2000s bears reproduced the same results on summer range size reduction and range shift as the full sample comparison (see Results). These analyses support our comparative work between decades on the subpopulation of polar bears tracked in Baffin Bay.

#### 2.2.4 Monthly and seasonal kernel density estimates

We estimated the geographic area characterized by a high probability of use by collared AF polar bears in Baffin Bay using a fixed kernel density approach (Worton, 1989). Kernel density estimators provide a nonparametric probability of using a given point in space and are reliably used to define the utilization distribution, or home range, for marine and terrestrial wildlife (Kie et al., 2010). We calculated Gaussian bivariate normal kernel density estimates for each decade (*n* = 2), month (*n* = 12), and season (*n* = 3) for all bears in the sample. Kernel density estimates (KDEs) were calculated using the “bkde2D” function in “KernSmooth” R package (Wand, 1994; Wand & Jones, 1995). As the sample size of collared bears slightly differed between the 1990s and 2000s (Table 1), we generated random samples of equal size (*n* = 38) from the pool of AF bears in each decade. We sampled bears with replacement 1,000 times for each monthly and seasonal KDE and calculated the area of the 95% contour polygon (bounding 95% of the KDE surface volume). We produced a mean and bootstrapped standard error (SE) for monthly and seasonal home ranges, and used the “intersect” tool in ArcGIS to calculate overlap in home ranges between decades. We also estimated the proportion of home range overlap between the 1990s and the 2000s (Fieberg & Kochanny, 2005) based on the bootstrapped mean. We used a cell size of 6 km and bandwidth of 50 km (approximately 50% of the 4‐day movement step of AFs in this study) to calculated KDEs. We also examined whether there has been a distributional shift by calculating the median seasonal latitude values for AFs in the 1990s and 2000s of the KDE and of the pooled location data. We compared changes in median latitude and longitude across decades with *t* tests at a significance level of α = 0.05.

#### 2.2.5 Movements across subpopulation boundaries

Using each complete individual AF bear trajectory as a single sample, we calculated the number of days each bear spent in BB during the tracking period. We examined the departures from BB using telemetry data by estimating the fraction of bears that crossed BB management boundaries up to 300 days after capture. Specifically, we calculated the number of days since capture until each polar bear left BB or until the end of their observation period for those that were not observed to leave. For each decade, statistical methods for censored event times were used to construct “survival” curves (Kaplan–Meier) to characterize the distribution of first exit times, to estimate departure probabilities from BB, and test for differences among decades (log‐rank test of equality) with α = 0.05. We considered a departure to be at least 30 days long, thus bears that departed BB but returned in <30 days were not included in the estimates. We summarized which subpopulation bears departed to, the departure month, and compared this across decades. We also tested whether 1990s capture season (spring or fall) impacted the time until departure from BB by assessing capture season as a factor.

### 2.3 Harvest recoveries and recaptures of marked bears

We evaluated spatial patterns in live recaptures and dead recoveries of marked bears. We included bears that were marked in spring (April–May) or fall (August–October) during two periods when BB subpopulation mark–recapture studies were undertaken; 1991–1997 and 2009–2014. From 1991 to 1997, 881 polar bears were captured, physically marked, and tissue samples were collected (Taylor et al., 2005). Between 2011 and 2013, 1,111 unique individual bears were remotely biopsied (Pagano, Peacock, & McKinney, 2014) in western Baffin Bay, and between 2009 and 2013, 139 bears were physically captured or biopsied in eastern Baffin Bay (SWG, 2016; Table 2). During this second marking period (2009–2013), all sampled bears were genotyped to determine sex and individual identification. Since most bears in the 2000s were remotely biopsied, we also genotyped 635 of the 881 bears physically tagged in the 1990s that had neither been reported in the subsistence harvest in Nunavut or Greenland nor were >35 years old (maximum expected lifetime for polar bears) at the start of the genetic sampling in 2011 and were thus potentially alive. This allowed us to detect the number of bears sampled in the 2000s that had been originally physically marked in the 1990s.

**Table 2:** Number of genetic samples used from polar bears from the Baffin Bay (BB), Kane Basin (KB), Lancaster Sound (LS), and Davis Strait (DS) subpopulations for the winter‐spring analyses. For BB, 66 samples came from the subsistence harvest in the Baffin Island region (2011–2014) and 74 from biopsies taken in West Greenland by scientists during tagging operations (2009–2013)

	BB (2009–2014)	KB (2012–2014)	LS (2011–2013)	DS (2012–2013)	*N*
	Biopsy and Harvest	Biopsy	Harvest	Harvest	Total
Subadults	31	21	30	11	93
Adult females	54	54	15	11	134
Adult males	55	24	69	26	174
Adults combined	109	78	84	38	309
Total sample	140	99	114	49	402

Recapture or harvest recovery of physically or genetically marked individuals was detected by two means. Prior to 2011, when biopsy darting began in BB, marked individuals were identifiable by uniquely numbered ear tags and lip tattoos applied at first capture. Recaptures of these marked individuals were recorded during physical capture sampling in BB and surrounding subpopulations which took place 1991–1997 (BB, KB, and LS) and 2005–2007 (DS). Baffin Bay and adjacent subpopulations (KB, DS, LS) support a subsistence harvest of polar bears, which is characterized by high hunter reporting and management strategies designed to achieve maximum sustainable yield based on demographic data estimated from capture–recapture studies (SWG, 2016). Harvest recoveries of marked bears were detected via hunter returns of ear tags and lip tattoos as part of the continuous harvest monitoring program across all subpopulations in Canada and Greenland (Peacock, Laake, Laidre, Born, & Atkinson, 2012), though over time tag loss leads to lower probability of detection of recapture of physically marked individuals. Genetic identifications of individuals were analogous to the physical tags; however, genetic marks do not suffer from tag loss. The recapture or harvest recovery of marked individuals was detected by the presence of physical marks or by matching genetic ids.

We summarized the frequencies and proportions of marked bears that were recaptured or recovered inside versus outside BB to provide a crude assessment of the fidelity of marked bears to the BB subpopulation. With respect to short‐term fidelity, the frequencies of harvest recovery of individuals marked between 2011 and 2013 were examined to assess whether bears sampled during the recent BB mark–recapture study (SWG, 2016) remained within the subpopulation during the sampling interval. For this assessment, all recoveries were detected by matching genotypes of harvested bears to genotypes of marked individuals. A few of these individuals were also detected by hunter returns of tags and tattoos.

We also updated previously reported information on long‐term fidelity and patterns of harvest recovery in BB (Taylor & Lee, 1995; Taylor et al., 2001; Peacock et al., 2012) by examining the frequencies of bears originally marked in the 1990s that were recovered in the harvest between 1991 and 2014. Recoveries were detected via several means during this 14‐year monitoring period. From 1991 to 2010, recoveries were detected solely by hunter returns of tags and lip tattoos from marked bears. From 2011 to 2014, recoveries were detected either by tag and tattoos or by matching the genotype of a harvested bear to the genotype of one of the 635 marked individuals that could theoretically have been alive in 2011.

Finally, we used mark–recapture data from 1991 to 1997 and 2011 to 2013 in BB together with over 1,500 physical captures of polar bears in the neighboring DS subpopulation between 2005 and 2007 (Peacock, Taylor, Laake, & Stirling, 2013) to summarize recapture frequencies. Given the loss of sea ice that has occurred in both areas (Stern & Laidre, 2016) and the opportunity presented by having three intensive periods of mark–recapture that were relatively close in time, we used these data to assess movement between these two subpopulations. In particular, we examined whether there was evidence that DS bears had moved northward, in concert with sea‐ice loss, across the BB‐DS subpopulation boundary. For this assessment, recaptures were detected by several means. Recaptures of bears originally marked in BB 1991–1997 were detected during sampling in DS (2005–2007) by observations of tags and lip tattoos. Recaptures of bear marked in DS (2005–2007) were detected during sampling in BB (2011–2013) by matching genotypes.

### 2.4 Genetic methods

We used a contemporary subsample of genetic data from 402 biopsied, physically captured, and harvested polar bears sampled during the winter and spring (November–May 2009–2014) representing polar bears from four neighboring subpopulations: BB, KB, LS, and DS (Figure 1, Table 2). We used samples collected only within these two seasons because bears are widely distributed within their purported management boundaries and it excludes displacement of large groups of polar bears during the ice‐free season (see Appendix S1). While these samples were collected and analyzed primarily for a genetic mark–recapture (MR) assessment (SWG, 2016), the eight polymorphic microsatellites selected were used in population genetic analyses with the objective of assessing differentiation between BB and neighboring subpopulations. As the samples were recent and temporally congruent, these analyses provided an update to past population genetic studies (Paetkau et al., 1999; Peacock et al., 2015; Malenfant, Davis, Cullingham, & Coltman, 2016) that used samples from the 1990s. We used standardized population genetic analytical tools and methods to investigate genetic connectivity between subpopulations (ADEGENET package, Jombart, 2008; ARLEQUIN Version 3.5.1, Excoffier & Lischer, 2010; BA3‐3.0.3, Wilson & Rannala, 2003; DAPC, Jombart, Devillard, & Balloux, 2010; FSTAT, Goudet, 1995; GENECLASS2, Piry et al., 2004; GENELAND, Guillot, Mortier, & Estoup, 2005; Guillot, 2008; STRUCTURE, Pritchard, Stephens, & Donnelly, 2000). Genetic differentiation between groups was analyzed using unbiased *F*_ST_ statistics (Weir & Cockerham, 1984) in ARLEQUIN Version 3.5.1 (Excoffier & Lischer, 2010). The degree of population differentiation was analyzed after 10,000 permutations to estimate the genetic variability between subpopulations (see Appendix S1).

## 3 RESULTS

### 3.1 Radio telemetry

**Figure 2:**
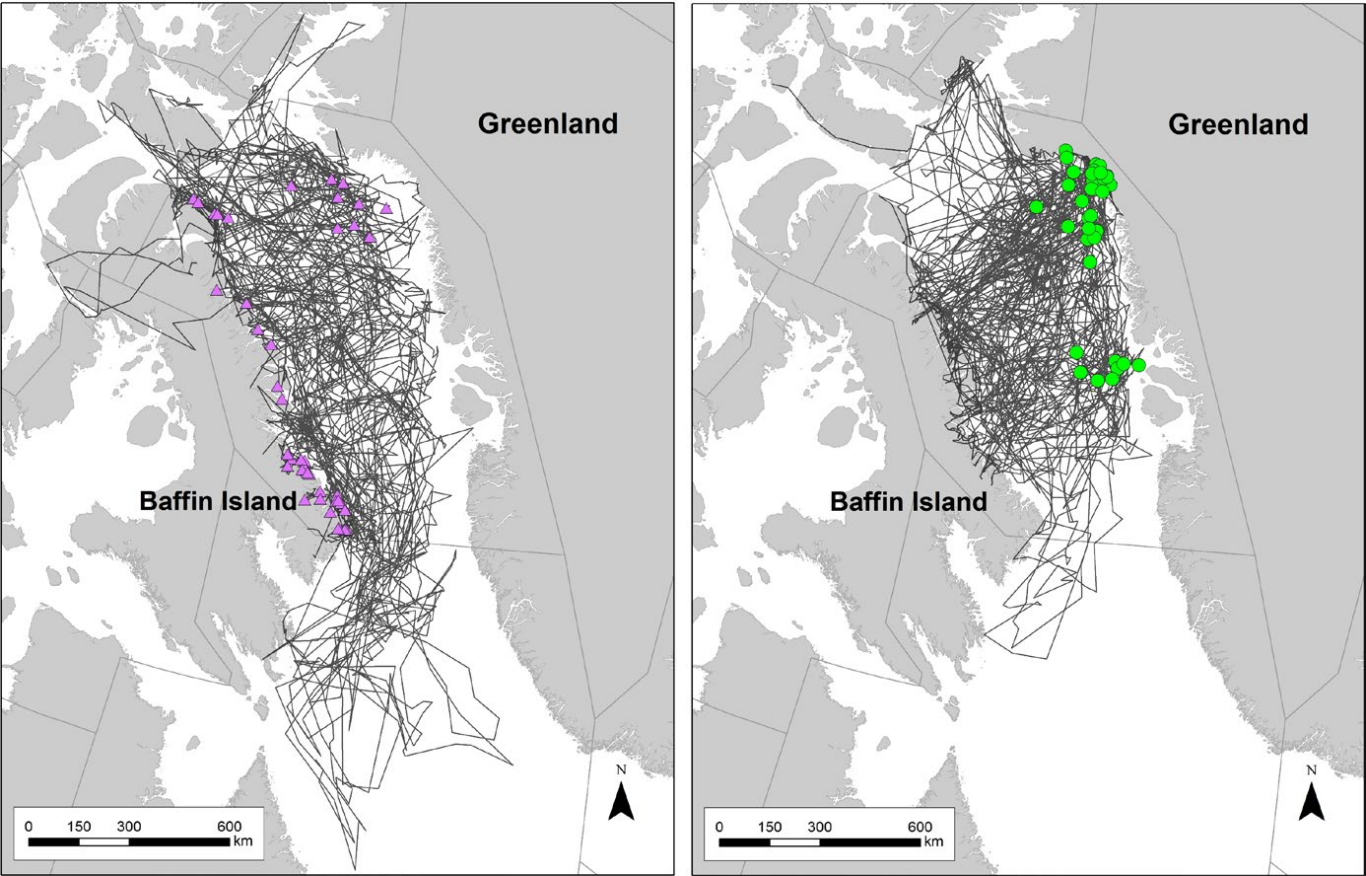
Movements of adult female polar bears collared in Baffin Bay in the 1990s (left) and 2000s (right). Colored symbols show locations where the bears were captured (cf. Figure 1)

Adult females in all reproductive states were fitted with collars in the 1990s (*n* = 43) and 2000s (*n* = 38) (Table 1). Collars deployed between 1991 and 1995 transmitted for an average of 438 days (*SD* 98), through 1997. Collars deployed between 2009 and 2013 transmitted for an average of 662 days (*SD* 346), through April 2015 (Figure 2). In the 1990s, 31 bears (72% of the total sample) were collared in fall on land on Baffin Island, 6% on the sea ice off the Baffin Island coast, and 21% on the sea ice in West Greenland. Due to a shift away from immobilization of wildlife for research studies in Nunavut, Canada after the 1990s, all collaring of adult females in the 2000s occurred on the spring sea ice in West Greenland. Polar bears in both decades ranged over the entire Baffin Bay during the ice‐covered season, freely moving back and forth between Baffin Island and West Greenland, though the disappearance of the sea‐ice platform in the southern extent of the range prevented long‐distance movements into Davis Strait in the 2000s (Figure 1).

### 3.2 Subpopulation ranges

**Figure 3:**
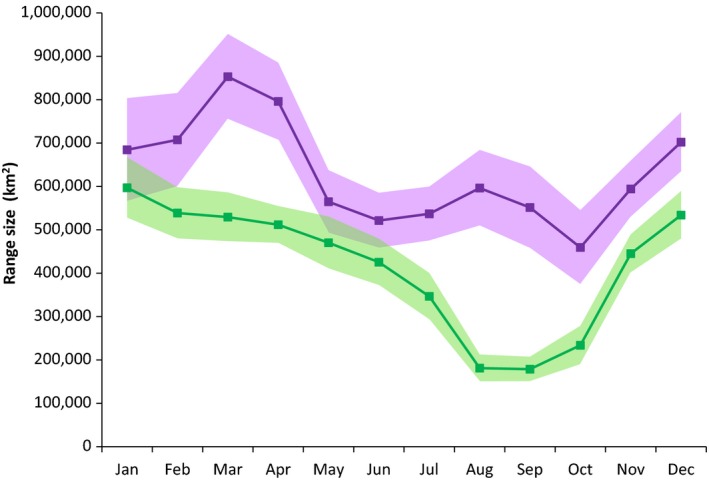
95% mean kernel range sizes (km^2^) estimated from a bootstrap method for adult female polar bears in Baffin Bay by decade and month (1990s purple; 2000s green). Line represents the mean values and shaded area represents ±2 SE; thus, lack of overlap between shaded areas indicates statistical significance. Graph excludes year‐round residents in Melville Bay, West Greenland

**Figure 4:**
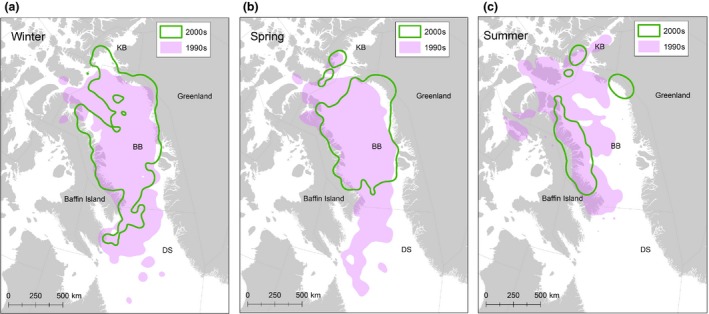
95% bivariate kernel ranges for adult female Baffin Bay polar bears captured in the 1990s and 2000s by season (a ‐ winter, b ‐ spring, c ‐ summer)

In the 1990s, monthly 95% bivariate kernel range sizes varied between 460,000 and 805,000 km^2^ (Figure 3) Monthly range sizes in the 2000s varied between 178,000 and 600,000 km^2^ (Figure 3) and were significantly smaller in all months except January, May, and June. Seasonal range sizes also exhibited significant reductions between the 1990s and 2000s (Figure 4). During the on‐ice seasons, there was a 17% range reduction in winter (1990s = 906,657 km^2^, 2000s = 593,077 km^2^, *p* = .012, Figure 4a) and a 30% range reduction in spring (1990s = 837,036 km^2^, 2000s = 593,077 km^2^, *p* < .001, Figure 4b). During the ice‐free season in summer, we found a 70% reduction in range size (1990s = 716,767 km^2^, 2000s = 211,476 km^2^, *p* < .001, Figure 4c). To evaluate whether the substantial summer range reduction was influenced by capture location, we compared range sizes between a subsample of the 1990s bears collared in Melville Bay, West Greenland on the spring pack ice (*n* = 9), and the full sample from the 2000s. We found similar patterns; the subsample of bears in the 1990s also used significantly larger ranges in summer (i.e., Figure 4c), and this resulted in a > 35% summer range reduction for the 2000s (*p* < .001).

**Figure 5:**
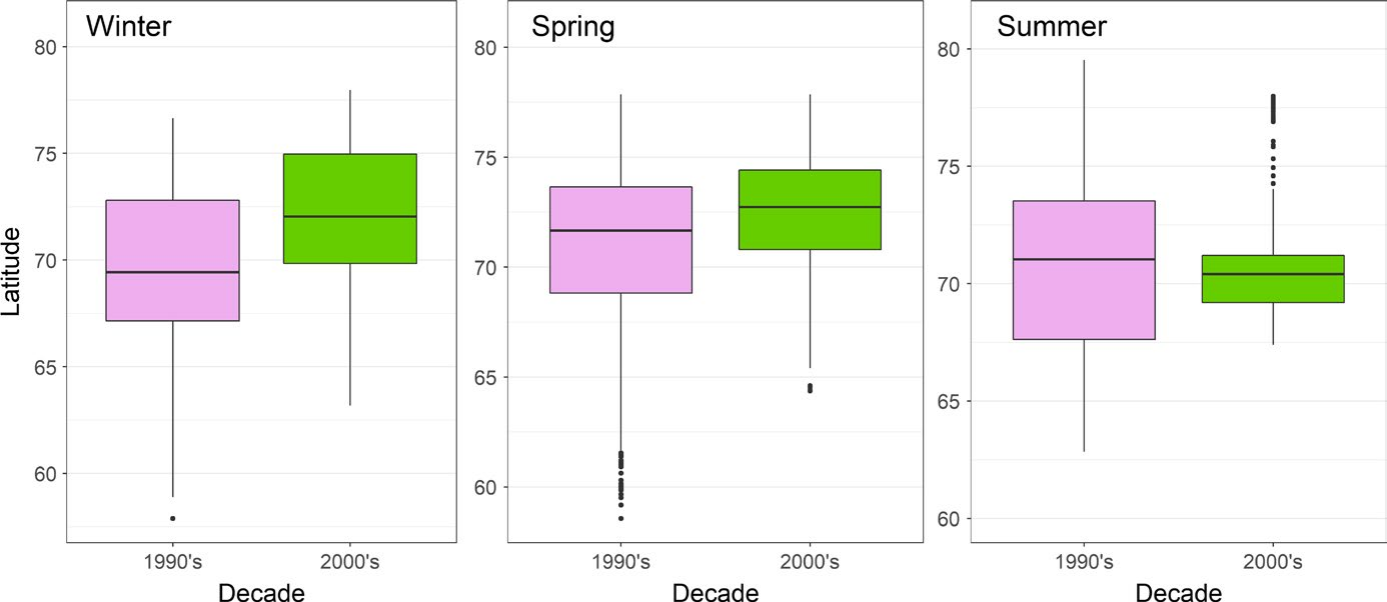
Box plots and interquartile range (shaded area) of median latitude of adult female polar bears in Baffin Bay by season in the 1990s and 2000s shown for each season. Plots exclude year‐round residents in Melville Bay, West Greenland

The reduction in range sizes resulted in a reduced habitat area used by bears in the 2000s. There was <50% overlap in ranges for bears in the 1990s and 2000s in all months and seasons. Range overlap across decades was lowest in September and October (~30%) and higher in winter and spring (60–80%). Additionally, there was a significant northward shift in the median subpopulation latitude in both winter (1990s, median 69.4°N; 2000s, median 72.0°N 2000s; *p* < .001) and spring (1990s, median 71.7°N; 2000s, median 72.7°N; *p* < .001). There was no northward shift between decades during summer. However, there was a significant contraction around 70.5–71°N between the two decades (Figure 5). These same patterns were demonstrated by the analyses of the subsample using *n* = 9 bears from the 1990s.

### 3.3 Movement between subpopulations

Overall, a significantly lower fraction of collared polar bears left BB in the 2000s than in the 1990s (χ^2^ = 6.8, *df* = 1, *p* = .009). The reductions in numbers of bears departing BB across subpopulation boundaries were due to fewer bears moving south into DS and fewer bears moving west into LS. In the 1990s, 14 of 43 (33%) radio‐collared bears moved south into DS, and 12 of 43 (28%) bears moved west into LS. In the 2000s, movements occurred to these areas but at a significantly lower rate; three of 38 (8%) tagged bears moved to DS, and three of 38 (8%) tagged bears moved to LS. In the 2000s, five of 38 (13%) bears also moved north directly from BB into KB, and an additional two moved to KB after first moving to LS. In the 1990s, *n* = 2 of 43 bears moved to KB and both initially moved into LS.

**Figure 6:**
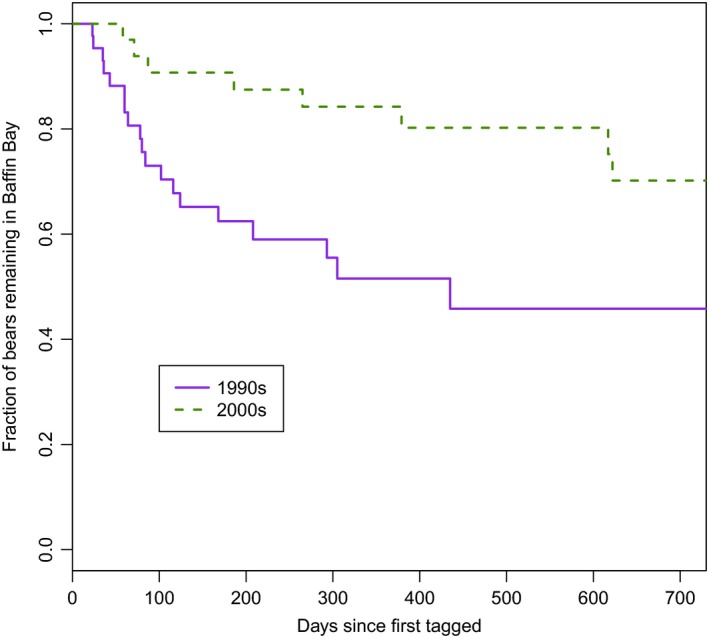
Percent of polar bears remaining in Baffin Bay after capture (1990s and 2000s) as a function of time. Departures are for areas outside of the BB subpopulation management boundaries. Only departures >30 days were considered

At 100 days after capture, approximately 73% (95% CI: 60%–88%) of collared bears remained in BB in the 1990s, whereas in the 2000s 91% (95% CI: 81%–100%) remained (*Z* = 2.041, *p* = .041) (Figure 6). At 300 days after capture, approximately 55% (95% CI: 43%–74%) of collared bears remained in BB in the 1990s, whereas in the 2000s, 84% (95% CI: 72%–98%) remained (*Z* = 2.732, *p* = .006; Figure 6). Overall, 56% of the collared bears never left BB during the 1990s tracking period, whereas 79% never left in the 2000s tracking period (Table 3). Individuals departed and returned several times over the course of the tracking period. Formal estimation of rates of return was complicated by highly variable follow‐up times; however, bears generally moved back and forth more frequently between BB and DS in the 1990s. Results indicated that capture season in the 1990s (spring vs. summer) did not influence timing of departure (*p* = .562).

**Table 3:** Number of subpopulation boundary crossings made by individual radio‐collared adult female bears in Baffin Bay in 1990s (*n* = 43) and the 2000s (*n* = 38) for departures of >30 days. Percentages shown as percent of total collared bears

	Number of subpopulation boundary crossings							
	0	1	2	3	4	5	6	7
1990s	24 (56%)	8 (19%)	6 (14%)	1 (2%)	1 (2%)	1 (2%)	1 (2%)	1 (2%)
2000s	30 (79%)	3 (8%)	2 (5%)	0	1 (3%)	1 (3%)	0	1 (3%)

The timing of departures over the annual cycle varied with patterns of seasonal ice coverage in BB. Bears were significantly more likely to leave and move south into DS during the winter or early spring (November through April) when DS was covered with pack ice (*p* < .001, Fisher's exact test). During June through September, bears in the 1990s were more likely to move into LS where summer remnants of sea ice were available in the archipelago (*p* = .003, Fisher's exact test). This summer movement pattern was only weakly present in the 2000s.

### 3.4 Harvest recoveries and recaptures of marked bears

Of the 1,250 bears marked in BB (2011–2013), 85 individuals were recovered in the reported subsistence harvest in Nunavut and Greenland or detected through genotyping during 2011–2014. Of these individuals, 84 (99%) were recovered in the BB harvest, and one bear was harvested in DS. Also during this period, 11 bears marked in the 1990s in BB were recovered in the harvest (all in BB).

Of the 881 bears marked in BB from 1991 to 1997, 181 individuals were recovered in the subsistence harvest in Canada and Greenland between 1991 and 2014; 83% of these recoveries occurred within BB. Recoveries of marked BB bears outside the bounds of the BB subpopulation tended to be male‐biased (3.29 males per female) relative to recoveries within BB (1.85 males per female), although this was not statistically significant (*p* = .483, Fisher's exact test).

Of the 1,518 unique individuals captured in DS in the mid‐2000s (Peacock et al., 2013), 13 (0.8%) were recaptures from the sample of 881 bears originally marked in BB between 1991 and 1997. Of the bears sampled in BB from 2009 to 2013 (1,250 unique individuals, 1,623 capture events), 16 (1%) were originally marked in DS between 2005 and 2007. In sum, from a total of 2,771 bears marked in either BB or DS during 2005–2013, 29 instances were detected where marked bears moved from one subpopulation to the other. Despite extensive marking of bears throughout the range of both subpopulations, the frequency of recorded interpopulation movements was relatively low and mainly clustered near the boundary. Based on straight line distance between capture location and the BB‐DS subpopulation boundary, the 29 individuals that made interpopulation movements were first captured significantly closer to the boundary than other bears marked in these subpopulations (χ^2^ = 169.48, *df* = 11, *p* < .001). Seventy percent of bears known to have made interpopulation movements were originally captured within 100 km of the boundary, compared to 20% for bears that did not make such movements.

### 3.5 Genetic connectivity between Baffin Bay and other subpopulations

The average expected heterozygosity, based on eight polymorphic microsatellites, for 402 polar bears sampled during winter–spring (November–May) in 2009–2014 ranged from 0.779 ± 0.018 (KB) to 0.804 ± 0.012 (BB and LS). No significant deviation from Hardy–Weinberg Equilibrium was observed in any of the datasets (Table S1) nor was a linkage disequilibrium observed between all pairs of loci within the dataset. Population structure estimated using multilocus *F*_ST_ statistics was generally low, although tests were statistically significant after sequential Bonferroni correction (Rice, 1989) at α = 5% level (Table 4). Pairwise *F*_ST_ values showed low but significant differentiation for BB between KB, DS, and LS when comparing the winter and spring samples for bears of all ages and sexes, adults of both sexes, and adult females (Table 4). There were no significant differences for adult males and subadults (both sexes). These results provide the first evidence for a genetic difference between BB and KB, in contrast to previous studies by Paetkau et al. (1999), Peacock et al. (2015), and Malenfant et al. (2016). The discriminant analysis of principal components (DAPC, Jombart et al., 2010), separated BB and KB from LS and DS, supporting the grouping observed using GENELAND (Figures S1–S4, Table 2). Of note, Bayesian clustering method in STRUCTURE did not separate BB and KB, possibly due to low F_ST_ values (see Appendix S1).

**Table 4:** Pairwise *F*_ST_ estimates testing for population structure across Baffin Bay (BB), Kane Basin (KB), Lancaster Sound (LS), and Davis Strait (DS) polar bear subpopulations

	BB	LS	DS
Subadults			
LS	0.003		
DS	0.012	0.018	
KB	0.01	0.008	**0.024**
Adult females			
LS	0.005		
DS	0.007	0.012	
KB	**0.008**	0.003	**0.022**
Adult males			
LS	**0.013**		
DS	0.009	**0.016**	
KB	0.003	**0.010**	**0.013**
Adults combined			
LS	**0.008**		
DS	**0.01**	**0.015**	
KB	**0.006**	**0.013**	**0.016**
All Bears			
LS	**0.007**		
DS	**0.009**	**0.014**	
KB	**0.005**	**0.012**	**0.015**

Bold value is significant at *p* < .05 after sequential Bonferroni correction (Rice, 1989).

## 4 DISCUSSION

Over the past three decades, there has been a significant decline in sea ice in Baffin Bay and surrounding areas (Stern & Laidre, 2016), which has directly translated to loss of habitat available to the BB polar bear subpopulation. We document that BB subpopulation has become increasingly isolated through contraction of seasonal ranges and reductions in emigration across subpopulation boundaries. We suggest that this is a consequence of ongoing loss of sea ice in Baffin Bay and reflects climate impacts that are likely to occur for other subpopulations in the seasonal sea‐ice ecoregion (Amstrup et al., 2008).

### 4.1 Satellite telemetry

The reliability of inferences about population structure based on movement data is dependent on the extent to which sampled individuals represent the subpopulation of interest. This is a function of sampling strategy, timing, and sample size. In this study, we investigated the movement of AFs and interpret the data as representative for the subpopulation as a whole. The movements of individual polar bears have been studied using satellite telemetry for decades (e.g., Amstrup, Durner, Stirling, Lunn, & Messier, 2000; Born, Wiig, & Thomassen, 1997; Laidre et al., 2013, 2015; Wiig, Born, & Pedersen, 2003), and inference has almost exclusively been based on adult females because of the challenges of instrumenting adult males (Wiig et al., 2017). Our analyses, which also included harvest recoveries of both sexes, did not indicate vastly different movement between males and females. Previous studies in BB also indicated that both sexes utilize the same habitats in spring (Laidre et al., 2013). We therefore suggest our satellite telemetry data are a representative index of broad population movement patterns.

Our analyses addressed differences in sampling between decades to ensure that the 1990s and 2000s data, which were collected within the boundaries of BB but different areas and seasons, were comparable. Bears collared in West Greenland in the 2000s used nearly the entire Baffin Island coastline in fall and were distributed over the same capture region and same range of latitudes where females in the 1990s were collared, with the exception of the southern point around Cape Dyer. Further, subsample analyses of a small number of bears collared in spring in a restricted area of West Greenland in the 1990s (*n* = 9) provided the same significant results on summer range reduction as the larger dataset. Overall, in our study, sample sizes for each decade were roughly equivalent (1990s, *n* = 43; 2000s, *n* = 38), sampling durations were similar (6‐ to 7‐year tracking periods in each decade), and collars in both decades transmitted up to 3 years. Finally, in both decades, collar deployments were distributed over multiple years and over broad geographic areas within BB management boundaries (Figure 1).

We found that seasonal ranges of adult females in BB became significantly smaller, by a third to a half, between the 1990s and 2000s. This range contraction coincided with a northward shift during the on‐ice season. There was no northward shift in summer because bears’ ranges contracted on land around similar latitudes as used in the 1990s. Further there was <50% overlap in habitats used in the 1990s and the 2000s. The same patterns and statistical significance were found when a small fraction of Greenland resident bears (AFs that remain year‐round at Melville Bay glacier fronts, SWG, 2016) were excluded from the analyses. Satellite telemetry also demonstrated significant reduction in emigration from BB since the 1990s. Finally, microsatellite analyses for the first time indicated a weak but significant differentiation between adults in BB and KB. Harvest recoveries suggest fidelity to BB has remained high both across and within decades. Our results indicate the BB subpopulation has become more discrete, with less exchange between other neighboring subpopulations. These changes are likely due to reduced sea‐ice extent in winter, early break‐up in spring (Stern & Laidre, 2016), and complete absence of sea ice in summer (SWG 2016).

### 4.2 Recovery of marks

Although the use of tag recoveries is a relatively coarse means of assessing subpopulation closure, it can facilitate the inclusion of data from large numbers of individuals and be complementary to telemetry. The probability of detecting interpopulation movements depends on many factors, including the number of initial marks deployed, the persistence and probability of detecting marks, and the intensity of harvest to recover marks. Additionally, the detection of movement amongst subpopulations does not provide a means of quantifying rates of permanent emigration or immigration. Nevertheless, data on harvest recoveries of marked bears provide a supplemental line of evidence to support subpopulation delineations based on more detailed methods such as cluster analysis of movement data (Taylor et al., 2001).

We documented very low levels of harvest recovery of bears outside their subpopulation of origin based on data collected between 2011 and 2014. Bears marked in the final year of study had a near zero probability of recovery because harvest monitoring was not extended far beyond the last year of marking (in 2013). However, bears marked in the first 2 years were available for recovery in the harvest, subject to rates of natural mortality. The total number of bears marked in 2011 and 2012 was equivalent to ~32% of the point estimate of abundance for the BB subpopulation (SWG, 2016). Despite marking a large proportion of the subpopulation and the continuous monitoring of harvest within BB and surrounding subpopulations, instances of emigration were only approximately 1% of the recoveries of BB marks. Our findings imply that BB bears exhibit a high degree of short‐term fidelity to this geographically defined unit, which is consistent with estimates of site fidelity derived from mark–recapture analyses (SWG, 2016). Examining long‐term harvest recoveries of marked bears over a 14‐year period, we found that 83% of recovered bears marked in BB in the 1990's were recovered within BB. Considering BB and adjacent subpopulations were subject to broadly similar harvest rates and management regimes during this interval, this suggests relatively high long‐term fidelity to BB (PBSG 2010). Of note, prior to 2006 when quotas were introduced in Greenland, there was significant uncertainty in the accuracy of the reported polar bear harvest from BB and KB (SWG, 2016). The extent to which marked bears were under or over reported prior to that date is unknown, however, new monitoring and mandatory reporting laws have substantially improved reporting since then.

For comparison with our results, recent demographic analyses for BB used live‐recapture–dead‐recovery models to estimate probabilities of natural survival, harvest mortality, and fidelity in a unified modeling framework (SWG, 2016), with estimated time‐constant fidelity rates of 0.97 for males age ≥2 years, and 0.96 for other sex and age classes for the period 1993–2013. Although sample sizes were insufficient to investigate complex patterns and “fidelity” was defined relative to the onshore, summer sampling area (as opposed to the BB management unit, in the current analysis) results from capture–recapture modeling support our finding of relatively high fidelity of polar bears in BB.

### 4.3 Movements between Baffin Bay and Davis Strait

The boundary between the BB and DS subpopulations is spanned by pack ice in spring that provides a continuous platform for bears to move between subpopulations (Stern & Laidre, 2016). Nevertheless, consistent differences between BB and DS polar bears have been shown through genetics (Paetkau et al., 1999; Peacock et al., 2015; this study), movements (Taylor et al., 2001), and diet (Thiemann, Iverson, & Stirling, 2008), suggesting a functional boundary between them. Here, we investigated if there has been an increased exchange of polar bears across this boundary, which could contribute to exchange between subpopulations. Since 1990, marking effort (i.e., number of unique individuals marked) in BB and DS was equivalent to 41% (BB 1991–1997), 70% (DS 2005–2007), and 44% (BB 2011–2013) of estimated abundance at the time of marking (Peacock et al., 2013; SWG, 2016). The large number of marks released, combined with few interpopulation recaptures or recoveries between BB and DS (1% in each direction), suggest that the boundary between these two subpopulations remains relatively strong. Similarly, the telemetry data of collared BB bears demonstrate a reduced frequency of visitation to DS.

Bears that moved between BB and DS tended to be initially captured close to the BB‐DS boundary (<100 km). This may reflect the high degree of fidelity shown by BB and DS bears to their seasonal onshore range (e.g., Taylor et al., 2001), similar for other subpopulations (Wiig, 1995; Mauritzen, Derocher, & Wiig, 2001; Stirling, Lunn, Iacozza, Elliott, & Obbard, 2004; Zeyl, Ehrich, Aars, Bachmann, & Wiig, 2010).

### 4.4 Genetic connectivity between subpopulations

Previous analysis of polar bear population structure using microsatellites showed no significant genetic differences between BB and neighboring KB (Paetkau et al., 1999), though studies have found that bears from BB‐KB differed genetically from the LS and DS subpopulations (Paetkau et al., 1999; Peacock et al., 2015; Malenfant et al., 2016). In contrast, we found low but significant F_ST_ estimates between winter–spring samples from BB and KB. This could be due to groupings of closely related individuals (e.g., siblings or parent‐offspring in the samples). However, another explanation is that the results may be an early indication of increasing differentiation between the two subpopulations. This pattern was not captured by the Bayesian methods due to the low observed *F*_ST_ estimates as Bayesian STRUCTURE does not always infer the correct number of clusters when *F*_ST_ < 0.02 (Chen, Durand, Forbes, & François, 2007; Latch, Dharmarajan, Glaubitz, & Rhodes, 2006). Our study provides an updated genetic comparison between BB and KB based on recent data collected after the onset of large‐scale sea‐ice loss in Baffin Bay and Kane Basin. In all prior studies, the KB samples were collected in the early 1990s (Paetkau et al., 1999; Peacock et al., 2015; Malenfant et al., 2016).

Future sea‐ice loss is expected to fragment polar bear subpopulations, increase isolation, alter gene flow, and disrupt population boundaries (Derocher, Lunn, & Stirling, 2004; Kutschera et al., 2016; Sahanatian & Derocher, 2012). Genetic variation in polar bears is relatively low, and genetics is a relatively insensitive means of identifying, defining, or detecting change in subpopulation structure, especially when compared to individual‐based telemetry and tag recoveries. Time‐lag effects in genetic composition are influenced by the relatively long generation length for polar bears (~11.5 years; Regehr et al., 2016), and several generations may be needed to demonstrate increased genetic isolation as a consequence of climate‐induced range contraction.

Collectively, our findings from multiple lines of evidence suggest that the current BB subpopulation boundaries continue to be relevant for harvest management and subpopulation monitoring. However, an important consideration is that the subpopulation range is contracting and there appears to be reduced connectivity with subpopulations to the west and south. Our results provide evidence that the BB subpopulation is becoming increasing isolated, with reduced range sizes, shifts northward, and reduced emigration across subpopulation boundaries. These changes are almost certainly driven by loss of sea ice (Stern & Laidre, 2016). In general, telemetry and genetics share major advantages including standardized objective methodologies for delineating demographic structure allowing for broad inferences on movements and for calculating immigration and emigration rates. In our study, both telemetry and tag recovery data provided early signals of range shifts and changes in individual movements between local populations. Although our genetic analyses had relatively coarse resolution, primarily because the markers used were intended for mark–recapture and not dispersal, the results indicated a differentiation between the BB and KB subpopulations. More detailed genetic analyses would be worthwhile to explore this further. Sea‐ice loss is predicted to continue and information from telemetry and marked or recovered bears through long‐term monitoring programs (i.e., Laidre et al., 2015) will be critical to understanding increased fragmentation among polar bear subpopulations.

One key question unanswered is how loss of sea ice will alter dispersal patterns of apex predators, either by increasing genetic exchange as barriers to dispersal are removed or by increasing isolation as platforms for genetic transfer disappear. In some cases, different patterns may emerge within a species, depending on their dispersal ability and how loss of habitat redistributes subpopulations. For example, in areas around the Arctic Basin where sea ice converges or diverges (Amstrup et al., 2008), polar bears from multiple subpopulations may become aggregated or even mix. However, in the seasonal ice polar bear ecoregion (Amstrup et al., 2008), where annual sea ice exerts strong control on movements and directly influences the amount of time and area bears have to use and interact, the loss of sea ice is likely to make subpopulations more isolated. These findings and methods may prove useful for reassessing polar bear subpopulation boundaries in a future with reduced sea ice. Changes in exchange between subpopulations are expected with climate change and broadly the concept of boundaries may have to be reassessed.

## CONFLICT OF INTEREST

None declared.

## AUTHORS CONTRIBUTION

EWB, KLL, NJL, SNA, and ØW conceived and designed the study and sampling scheme. EWB, MD, and SNA acquired permits. EWB, KLL, MD, SNA, and ØW conducted the fieldwork. KLL, LWA, PH, RMG, and SNA analyzed the data. All authors contributed to writing of the manuscript and approved the final published version.
