# Supplementary material for: Range contraction and increasing isolation of a polar bear subpopulation in an era of sea‐ice loss
Source: Ecol Evol. 2018 Jan 18;8(4):2062–75. doi: 10.1002/ece3.3809 (PMC5817132; doi:10.1002/ece3.3809)
Supplement: Supplementary file 1 [file ECE3-8-2062-s001.docx]

**Supplementary Material for Laidre et al.**

**Appendix S1. Material and Methods**

Dried biopsy samples and harvest specimens (frozen or in ethanol) were analyzed by Wildlife Genetics International (Nelson, B.C., Canada) using protocols previously validated for bears (Kendall et al. 2009). DNA was extracted from ~ 3mm^2^ pieces of tissue with QIAGEN DNeasy Blood and Tissue Kits (<http://www.qiagen.com/>). Most samples consisted of a plug of skin and sub-cutaneous tissue, which provided ample material for DNA extraction and residual tissue for future analyses. In a small proportion of cases, the available sample consisted of a tuft of hair. DNA was extracted from hair samples using approximately 10 guard hair roots or 30 pieces of underfur. In a few cases, where a biopsy sample contained no visible tissue, DNA was successfully extracted by soaking the barbed needle from the biopsy dart in the lysis mix (QIAGEN buffer ATL + proteinase K). The samples from the harvest consisted of hair, fat or muscle tissue from various parts of the body.

For population genetic analysis we used the 8 most variable microsatellite markers (*G10B, CXX20, G10H, G10P, 145P07, MU50, MU59, G10X)* chosen based on the required standard for marker variability (HE = 0.80; Paetkau 2003) for individual identity. Markers provided a suitable dataset for mark-recapture analysis yet were also examined for analysis of population structure. Markers were chosen based on allele frequency data from 1,771 polar bears for which complete 20-locus genotypes existed before the genetic mark-recapture study began to select markers for the analysis of individual identity (Government of Nunavut, unpublished data). We ranked the 20 microsatellite markers in the dataset by expected heterozygosity. In addition to individual identification by the 8 microsatellite markers, we determined sex for every sample, using a 9^th^ marker, or a *ZFX*/*ZFY* marker system. This 9th marker roughly halved the match probability (assuming a balanced sex ratio), even for close relatives, as well as provided replication of sex data for individuals that were sampled more than once.

The analysis of individual identity followed a 3-phase approach. Phase 1 was a first pass of all extracted samples using the 9 selected markers. Samples that failed at >6 of 9 markers on the first pass did not proceed further in the analyses. Previous experience has shown that such samples are prone to errors and run out of DNA before generating a complete (Phase 2) and reproducible (Phase 3) genotype (D. Paetkau, pers. comm.). The first pass was followed by a cleanup phase in which data points that were weak or difficult to read the first time were re-analyzed. During cleanup we used 5 µL of DNA per reaction instead of the 3 µL used during first pass. At the conclusion of the cleanup phase, the remaining samples (99.5%) had high-confidence scores for all 9 markers. In cases where the genetic sex result contradicted the reported sex based on field assessment, genetic sex was checked using a second independent sex marker (*amelogenin*; http://www.ncbi.nlm.nih.gov/pubmed/7695123), confirming the results and ruling out the possibility that a mutation at a particular marker was to blame. In all cases, results from the second marker confirmed that the field data was the source of error. The third and final phase of analysis was error-checking, following the published protocol of reanalyzing the mismatching markers in highly similar pairs of genotypes (Paetkau 2003).

**Table S1.** Genetic diversity for all groups analyzed in this study in terms of observed (H_o_) and expected (H_e_) heterozygosity, Allele richness (AR), deviations from HWE (F_IS_) and number of individuals (N) in the four management areas (FSTAT, Goudet 1995, GenAlEx, Peakall & Smouse 2006, 2012).

| **All bears** |  |  |  |  |  |  |  |  |  |  |
| --- | --- | --- | --- | --- | --- | --- | --- | --- | --- | --- |
| Locality | Variable | REN145 | P0G10B | CXX20 | MU50 | G10H | MU59 | G10P | G10X | Average |
| Baffin Bay | H_o_ | 0.800 | 0.757 | 0.764 | 0.786 | 0.821 | 0.850 | 0.757 | 0.807 | 0.793±0.012 |
|  | H_e_ | 0.787 | 0.815 | 0.768 | 0.835 | 0.794 | 0.841 | 0.750 | 0.842 | 0.804±0.012 |
|  | AR | 6.948 | 6.727 | 7.154 | 7.927 | 10.324 | 9.233 | 7.549 | 9.299 |  |
|  | F_IS_ | -0.017 | 0.071 | 0.005 | 0.059 | -0.035 | -0.01 | -0.01 | 0.041 | 0.014 |
|  | N | 140 | 140 | 140 | 140 | 140 | 140 | 140 | 140 |  |
|  |  |  |  |  |  |  |  |  |  |  |
| Lancaster Sound | H_o_ | 0.833 | 0.833 | 0.658 | 0.842 | 0.825 | 0.851 | 0.719 | 0.816 | 0.797±0.025 |
|  | H_e_ | 0.807 | 0.810 | 0.735 | 0.833 | 0.809 | 0.841 | 0.774 | 0.823 | 0.804±0.012 |
|  | AR | 6.998 | 6.673 | 5.998 | 8.974 | 10.569 | 8.89 | 8.203 | 8.944 |  |
|  | F_IS_ | -0.033 | -0.029 | 0.105 | -0.01 | -0.019 | -0.012 | 0.071 | 0.009 | 0.009 |
|  | N | 114 | 114 | 114 | 114 | 114 | 114 | 114 | 114 |  |
|  |  |  |  |  |  |  |  |  |  |  |
| Davis Strait | H_o_ | 0.816 | 0.694 | 0.857 | 0.918 | 0.653 | 0.837 | 0.796 | 0.918 | 0.811±0.034 |
|  | H_e_ | 0.811 | 0.789 | 0.781 | 0.835 | 0.717 | 0.844 | 0.781 | 0.869 | 0.803±0.017 |
|  | AR | 6 | 6 | 7 | 9 | 9 | 10 | 10 | 9 |  |
|  | F_IS_ | -0.007 | 0.121 | -0.099 | -0.101 | 0.09 | 0.009 | -0.019 | -0.058 | -0.01 |
|  | N | 49 | 49 | 49 | 49 | 49 | 49 | 49 | 49 |  |
|  |  |  |  |  |  |  |  |  |  |  |
| Kane Basin | H_o_ | 0.828 | 0.818 | 0.707 | 0.758 | 0.788 | 0.869 | 0.727 | 0.697 | 0.774±0.022 |
|  | H_e_ | 0.800 | 0.814 | 0.717 | 0.824 | 0.765 | 0.851 | 0.707 | 0.750 | 0.779±0.018 |
|  | AR | 6.999 | 6.495 | 5.999 | 8.178 | 9.795 | 9.401 | 8.237 | 8.852 |  |
|  | F_IS_ | -0.035 | -0.005 | 0.015 | 0.081 | -0.03 | -0.021 | -0.029 | 0.071 | 0.006 |
|  | N | 99 | 99 | 99 | 99 | 99 | 99 | 99 | 99 |  |
|  |  |  |  |  |  |  |  |  |  |  |
| **All Adults** |  |  |  |  |  |  |  |  |  |  |
| Locality | Variable | REN145 | P0G10B | CXX20 | MU50 | G10H | MU59 | G10P | G10X | Average |
| Baffin Bay | H_o_ | 0.826 | 0.743 | 0.789 | 0.807 | 0.817 | 0.817 | 0.734 | 0.789 | 0.790±0.012 |
|  | H_e_ | 0.792 | 0.817 | 0.775 | 0.827 | 0.809 | 0.836 | 0.753 | 0.841 | 0.806±0.011 |
|  | AR | 6.912 | 6.575 | 7.044 | 7.92 | 10.281 | 8.842 | 7.448 | 9.123 |  |
|  | F_IS_ | -0.043 | 0.091 | -0.018 | 0.024 | -0.009 | 0.023 | 0.025 | 0.063 | 0.02 |
|  | N | 109 | 109 | 109 | 109 | 109 | 109 | 109 | 109 |  |
|  |  |  |  |  |  |  |  |  |  |  |
| Lancaster Sound | H_o_ | 0.893 | 0.821 | 0.679 | 0.857 | 0.833 | 0.845 | 0.750 | 0.833 | 0.814±0.024 |
|  | H_e_ | 0.802 | 0.808 | 0.740 | 0.834 | 0.810 | 0.844 | 0.785 | 0.820 | 0.805±0.011 |
|  | AR | 6.986 | 6.427 | 5.983 | 8.959 | 10.166 | 8.519 | 8.237 | 8.519 |  |
|  | F_IS_ | -0.115 | -0.016 | 0.083 | -0.028 | -0.029 | -0.001 | 0.045 | -0.016 | -0.011 |
|  | N | 84 | 84 | 84 | 84 | 84 | 84 | 84 | 84 |  |
|  |  |  |  |  |  |  |  |  |  |  |
| Davis Strait | H_o_ | 0.816 | 0.658 | 0.816 | 0.895 | 0.605 | 0.789 | 0.868 | 0.921 | 0.796±0.039 |
|  | H_e_ | 0.807 | 0.760 | 0.793 | 0.826 | 0.715 | 0.846 | 0.805 | 0.859 | 0.801±0.017 |
|  | AR | 6 | 6 | 7 | 9 | 9 | 10 | 10 | 9 |  |
|  | F_IS_ | -0.011 | 0.136 | -0.029 | -0.084 | 0.155 | 0.067 | -0.08 | -0.073 | 0.007 |
|  | N | 38 | 38 | 38 | 38 | 38 | 38 | 38 | 38 |  |
|  |  |  |  |  |  |  |  |  |  |  |
| Kane Basin | H_o_ | 0.872 | 0.833 | 0.667 | 0.731 | 0.769 | 0.846 | 0.705 | 0.705 | 0.766±0.027 |
|  | H_e_ | 0.807 | 0.813 | 0.716 | 0.826 | 0.751 | 0.839 | 0.689 | 0.754 | 0.774±0.019 |
|  | AR | 6.994 | 5.999 | 5.992 | 7.42 | 9.079 | 9.286 | 7.217 | 8.686 |  |
|  | F_IS_ | -0.08 | -0.025 | 0.069 | 0.116 | -0.024 | -0.008 | -0.023 | 0.065 | 0.011 |
|  | N | 78 | 78 | 78 | 78 | 78 | 78 | 78 | 78 |  |
|  |  |  |  |  |  |  |  |  |  |  |
| **Subadults** |  |  |  |  |  |  |  |  |  |  |
| Locality | Variable | REN145 | P0G10B | CXX20 | MU50 | G10H | MU59 | G10P | G10X | Average |
| Baffin Bay | H_o_ | 0.710 | 0.806 | 0.677 | 0.710 | 0.839 | 0.968 | 0.839 | 0.871 | 0.802±0.035 |
|  | H_e_ | 0.768 | 0.805 | 0.742 | 0.855 | 0.741 | 0.858 | 0.751 | 0.844 | 0.796±0.018 |
|  | AR | 5.499 | 5.913 | 5.339 | 6.66 | 6.193 | 7.397 | 5.815 | 6.922 |  |
|  | F_IS_ | 0.078 | -0.002 | 0.089 | 0.172 | -0.134 | -0.131 | -0.119 | -0.033 | -0.009 |
|  | N | 31 | 31 | 31 | 31 | 31 | 31 | 31 | 31 |  |
|  |  |  |  |  |  |  |  |  |  |  |
| Lancaster Sound | H_o_ | 0.667 | 0.867 | 0.600 | 0.800 | 0.800 | 0.867 | 0.633 | 0.767 | 0.75 ±0.037 |
|  | H_e_ | 0.828 | 0.821 | 0.705 | 0.835 | 0.814 | 0.831 | 0.746 | 0.836 | 0.802±0.017 |
|  | AR | 6.554 | 6.126 | 5.359 | 6.798 | 7.702 | 6.835 | 5.275 | 7.122 |  |
|  | F_IS_ | 0.198 | -0.056 | 0.151 | 0.043 | 0.018 | -0.044 | 0.154 | 0.084 | 0.066 |
|  | N | 30 | 30 | 30 | 30 | 30 | 30 | 30 | 30 |  |
|  |  |  |  |  |  |  |  |  |  |  |
| Davis Strait | H_o_ | 0.818 | 0.818 | 1.000 | 1.000 | 0.818 | 1.000 | 0.545 | 0.909 | 0.864±0.054 |
|  | H_e_ | 0.823 | 0.835 | 0.701 | 0.853 | 0.723 | 0.866 | 0.680 | 0.866 | 0.793±0.028 |
|  | AR | 6 | 6 | 5 | 7 | 5 | 7 | 3 | 7 |  |
|  | F_IS_ | 0.006 | 0.022 | -0.457 | -0.183 | -0.139 | -0.164 | 0.205 | -0.053 | -0.094 |
|  | N | 11 | 11 | 11 | 11 | 11 | 11 | 11 | 11 |  |
|  |  |  |  |  |  |  |  |  |  |  |
| Kane Basin | H_o_ | 0.667 | 0.762 | 0.857 | 0.857 | 0.857 | 0.952 | 0.810 | 0.667 | 0.804±0.035 |
|  | H_e_ | 0.775 | 0.803 | 0.728 | 0.832 | 0.808 | 0.875 | 0.746 | 0.739 | 0.788±0.018 |
|  | AR | 5.38 | 6.244 | 5.647 | 6.773 | 6.457 | 7.553 | 6.611 | 5.8 |  |
|  | F_IS_ | 0.142 | 0.052 | -0.182 | -0.032 | -0.062 | -0.091 | -0.088 | 0.1 | -0.02 |
|  | N | 21 | 21 | 21 | 21 | 21 | 21 | 21 | 21 |  |
|  |  |  |  |  |  |  |  |  |  |  |
| **Adult females** |  |  |  |  |  |  |  |  |  |  |
| Locality | Variable | REN145 | P0G10B | CXX20 | MU50 | G10H | MU59 | G10P | G10X | Average |
| Baffin Bay | H_o_ | 0.796 | 0.741 | 0.852 | 0.833 | 0.852 | 0.778 | 0.870 | 0.778 | 0.813±0.016 |
|  | H_e_ | 0.796 | 0.814 | 0.791 | 0.840 | 0.797 | 0.833 | 0.785 | 0.852 | 0.814±0.009 |
|  | AR | 5.809 | 5.978 | 5.808 | 6.943 | 7.147 | 7.009 | 6.044 | 7.557 |  |
|  | F_IS_ | 0 | 0.091 | -0.077 | 0.008 | -0.069 | 0.067 | -0.109 | 0.088 | 0.001 |
|  | N | 54 | 54 | 54 | 54 | 54 | 54 | 54 | 54 |  |
|  |  |  |  |  |  |  |  |  |  |  |
| Lancaster Sound | H_o_ | 0.867 | 0.800 | 0.800 | 0.933 | 0.933 | 0.800 | 0.800 | 0.933 | 0.858±0.023 |
|  | H_e_ | 0.832 | 0.828 | 0.703 | 0.802 | 0.818 | 0.828 | 0.782 | 0.860 | 0.807±0.017 |
|  | AR | 6.604 | 5.967 | 5.45 | 7.791 | 7.993 | 6.459 | 6.388 | 7.434 |  |
|  | F_IS_ | -0.043 | 0.034 | -0.143 | -0.17 | -0.146 | 0.034 | -0.024 | -0.089 | -0.067 |
|  | N | 15 | 15 | 15 | 15 | 15 | 15 | 15 | 15 |  |
|  |  |  |  |  |  |  |  |  |  |  |
| Davis Strait | H_o_ | 0.909 | 0.818 | 0.909 | 1.000 | 0.545 | 0.909 | 0.818 | 0.727 | 0.830±0.050 |
|  | H_e_ | 0.818 | 0.810 | 0.827 | 0.844 | 0.814 | 0.887 | 0.874 | 0.818 | 0.837±0.010 |
|  | AR | 6 | 6 | 6 | 7 | 7 | 9 | 9 | 6 |  |
|  | F_IS_ | -0.117 | -0.011 | -0.105 | -0.196 | 0.341 | -0.026 | 0.067 | 0.116 | 0.009 |
|  | N | 11 | 11 | 11 | 11 | 11 | 11 | 11 | 11 |  |
|  |  |  |  |  |  |  |  |  |  |  |
| Kane Basin | H_o_ | 0.870 | 0.852 | 0.667 | 0.741 | 0.759 | 0.852 | 0.648 | 0.759 | 0.769±0.030 |
|  | H_e_ | 0.816 | 0.812 | 0.703 | 0.839 | 0.752 | 0.818 | 0.670 | 0.777 | 0.773±0.021 |
|  | AR | 6.37 | 5.744 | 5.272 | 6.537 | 6.144 | 7.005 | 5.517 | 6.935 |  |
|  | F_IS_ | -0.068 | -0.050 | 0.053 | 0.119 | -0.01 | -0.042 | 0.033 | 0.023 | 0.006 |
|  | N | 54 | 54 | 54 | 54 | 54 | 54 | 54 | 54 |  |
|  |  |  |  |  |  |  |  |  |  |  |
| **Adult males** |  |  |  |  |  |  |  |  |  |  |
| Locality | Variable | REN145 | P0G10B | CXX20 | MU50 | G10H | MU59 | G10P | G10X | Average |
| Baffin Bay | H_o_ | 0.855 | 0.745 | 0.727 | 0.782 | 0.782 | 0.855 | 0.600 | 0.800 | 0.768±.029 |
|  | H_e_ | 0.793 | 0.825 | 0.757 | 0.812 | 0.814 | 0.837 | 0.716 | 0.835 | 0.799±0.015 |
|  | AR | 6.78 | 5.995 | 6.367 | 7.33 | 9.484 | 8.081 | 7.134 | 8.547 |  |
|  | F_IS_ | -0.079 | 0.097 | 0.04 | 0.038 | 0.04 | -0.021 | 0.164 | 0.043 | 0.039 |
|  | N | 55 | 55 | 55 | 55 | 55 | 55 | 55 | 55 |  |
|  |  |  |  |  |  |  |  |  |  |  |
| Lancaster Sound | H_o_ | 0.899 | 0.826 | 0.652 | 0.841 | 0.812 | 0.855 | 0.739 | 0.812 | 0.804±0.027 |
|  | H_e_ | 0.798 | 0.807 | 0.736 | 0.841 | 0.811 | 0.840 | 0.790 | 0.816 | 0.805±0.012 |
|  | AR | 6.862 | 6.073 | 5.896 | 8.733 | 8.984 | 7.514 | 7.431 | 7.488 |  |
|  | F_IS_ | -0.127 | -0.024 | 0.115 | 0.001 | -0.001 | -0.018 | 0.065 | 0.005 | 0.001 |
|  | N | 69 | 69 | 69 | 69 | 69 | 69 | 69 | 69 |  |
|  |  |  |  |  |  |  |  |  |  |  |
| Davis Strait | H_o_ | 0.769 | 0.615 | 0.808 | 0.846 | 0.615 | 0.769 | 0.885 | 1.000 | 0.788±0.046 |
|  | H_e_ | 0.802 | 0.751 | 0.795 | 0.830 | 0.651 | 0.851 | 0.788 | 0.867 | 0.792±0.024 |
|  | AR | 5 | 5.995 | 6.918 | 8.842 | 8.837 | 8.918 | 9.76 | 8.918 |  |
|  | F_IS_ | 0.041 | 0.184 | -0.016 | -0.02 | 0.055 | 0.098 | -0.125 | -0.157 | 0.004 |
|  | N | 26 | 26 | 26 | 26 | 26 | 26 | 26 | 26 |  |
|  |  |  |  |  |  |  |  |  |  |  |
| Kane Basin | H_o_ | 0.875 | 0.792 | 0.667 | 0.708 | 0.792 | 0.833 | 0.833 | 0.583 | 0.760±0.035 |
|  | H_e_ | 0.776 | 0.826 | 0.748 | 0.800 | 0.760 | 0.865 | 0.739 | 0.696 | 0.776±0.019 |
|  | AR | 7 | 6 | 6 | 7 | 8 | 9 | 8 | 9 |  |
|  | F_IS_ | -0.131 | 0.043 | 0.111 | 0.116 | -0.043 | 0.038 | -0.13 | 0.165 | 0.021 |
|  | N | 24 | 24 | 24 | 24 | 24 | 24 | 24 | 24 |  |

**Figure S1 a and b.** Results of population and group structure explored using discriminant analysis of principal components (DAPC, Jombart et al. 2010), a multivariate method that uses the genetic relationship among individuals to identify groups. This was based on allele frequencies of the microsatellite markers and conducted using the *Adegenet* package (Jombart et al. 2008) in R (www.r-project.org; R Development Core Team 2008). Analyses used a contemporary sub-sample of genetic data from 402 biopsied, physically-captured, and harvested polar bears sampled during the winter and spring (Nov-May 2009-2014) representing polar bears from four neighboring subpopulations: BB, KB, LS and DS. We used samples collected during these two seasons as bears are widely distributed within their purported management boundaries and it excludes displacement of large groups of polar bears during the ice-free season. We assessed all bears in the Winter-Spring as well as Adults only. Figure A) Winter-Spring samples containing all samples from the defined period, b) Winter-Spring-Adult samples containing only the adults in the Winter-Spring sample (i.e. a sub-set of the entire Winter-Spring sample).

**
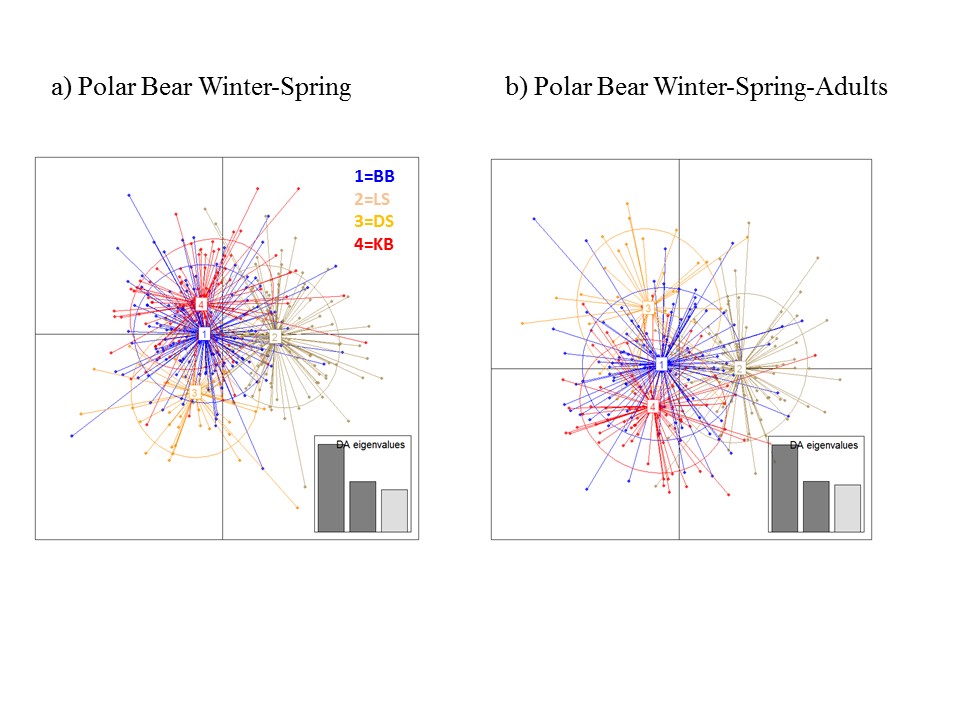
**

Two Bayesian clustering methods, STRUCTURE (Prichard et al. 2000) and GENELAND (Guillot et al. 2005b, Guillot 2008) were used to identify the most likely number of genetic different groups of polar bears and to assign the individuals to the identified clusters or groups. STRUCTURE groups individuals into clusters, minimizing deviation within clusters from Hardy–Weinberg equilibrium (HWE) and linkage equilibrium. GENELAND also used HWE and linkage disequilibrium but also included the spatial locations as prior information, thus assuming that neighboring individuals also are more closely genetically related, belonging to the same group/cluster (Guillot et al. 2009).

STRUCTURE analyses were performed using the admixture model and the model of correlated allele frequencies between clusters*.* The results of the tests were based on 1,000,000 iterations, 100,000 burn-in period and 10 independent runs. All samples were combined and assumed to have originated from one to five populations (=K) depending on the sample-unit without prior information of the sample’s origin and when using ‘locality’ as prior. We followed the recommendation made by Wang (2017) running STRUCTURE, adjusting ALPHA =0.25 according to the number of clusters expected (K=4 in this instance) to account for the unbalanced sample size. Number of clusters was inferred using ΔK (Evanno, Regnaut & Goudet, 2005) performed using STRUCTURE HARVESTER (Earl & VonHoldt, 2012). The software CLUMPAK that automatically processes STRUCTURE results across the independent runs of K was applied to visualize the results (Kopelman et al., 2015).

Analyses in GENELAND were conducted to infer the best number of genetic groups, K, during a run varying from one to six and 10 independent runs. The run parameters used a spatial uncertainty coordinate of 0.01, 500,000 Markov-Chain Monte Carlo (MCMC) iterations and a thinning factor of 100, using the Correlated Allele Frequency model and the spatial model based on the GPS coordinates for individuals. This created a map showing the probability of the group membership of the genetically different groups.

The results of STRUCTURE analyses indicated the existence of only one group in both WS or WSA data (results shown using CLUMPAK) and without ‘locality’ as a prior. Using ‘locality’ as prior ΔK (Evanno, Regnaut & Goudet, 2005) identified three clusters, BB-KB, LS and DS (Supplemental Figure 2 a, b, Supplementary Figure 3, a, b, c, d, e, f, g, h). The spatial information (GPS coordinates) implemented in GENELAND showed a group structure in all datasets, however with different number of groups (only WS and WSA results are shown). The heat-map of the posterior probabilities for the winter-spring polar bears dataset and the winter-spring - adult polar bear dataset both indicated the existence of four groups (Fig. 3). To verify whether these groups corresponded with the four different management groups, the origin of the individuals grouped by GENELAND were examined (Table 2). The two datasets identified one group containing almost all individuals from Baffin Bay and Kane Basin, a second group made up of all Lancaster Sound individuals, a third group involving almost all individuals from Davis Strait, and a fourth group comprising only individuals from Baffin Bay. A minor difference was observed amongst the distribution of Baffin Bay into the four groups using the WSA dataset but the overall pattern was identical to the groups of the WS dataset.

In conclusion, the Bayesian based methods based on individual GPS coordinates in GENELAND were able to identify four groups when using spatial information. However, the groups did not identify Baffin Bay and Kane Basin as genetically different groups supporting the findings suggested by Paetkau et al. 1999. Applying ‘locality’ as prior in STRUCTURE identified three clusters, BB-KB, LS and DS, again BB and KB was lumped as one unit. This can probably be attributed to the low F_ST_ estimates between the four management areas. Fx for STRUCTURE studies Latch et al. (2006) and Chen et al. (2007) have reported that a genetic differentiation of F_ST_ < 0.02 for the algorithm in STRUCTURE has problems inferring the number of clusters correctly. GENELAND is based on STRUCTURE and therefore an identical limit of genetic differentiation for group detection might be expected.

**Figure S2.** Graphical output from HARVESTER (2a Winter-Spring, b Winter-Spring-Adults) applying ΔK of Evanno et al. (2005) to infer the number of clusters and from STRUCTURE (Pritchard et al., 2000).

1. Winter-Spring


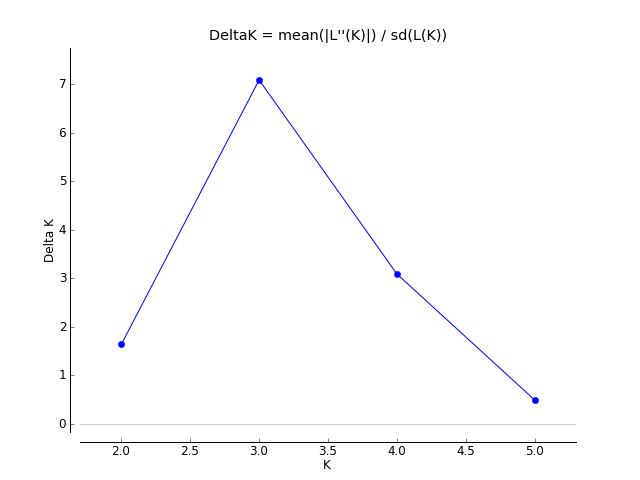


1. Winter-Spring-Adults


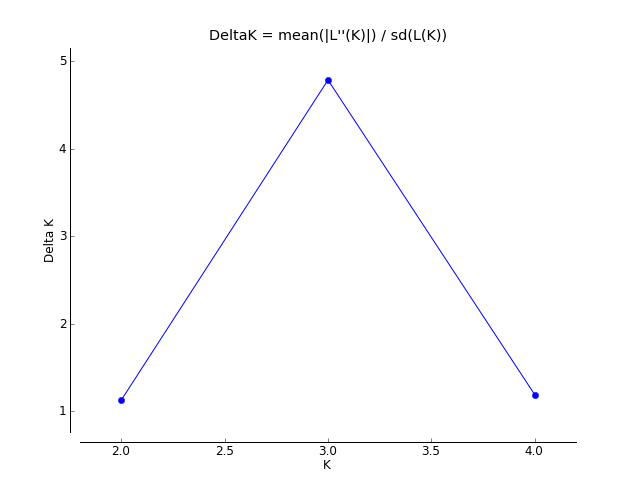


**Figure S3.** The results were processed in CLUMPAK (Kopelman et al., 2015) for WS (a-d) and WSA (e-h) with and without using ‘locality’ as prior for the different number of clusters (K) inferred. Each vertical line represents an individual, and the color composition shows the probability of belonging to each of the defined clusters.


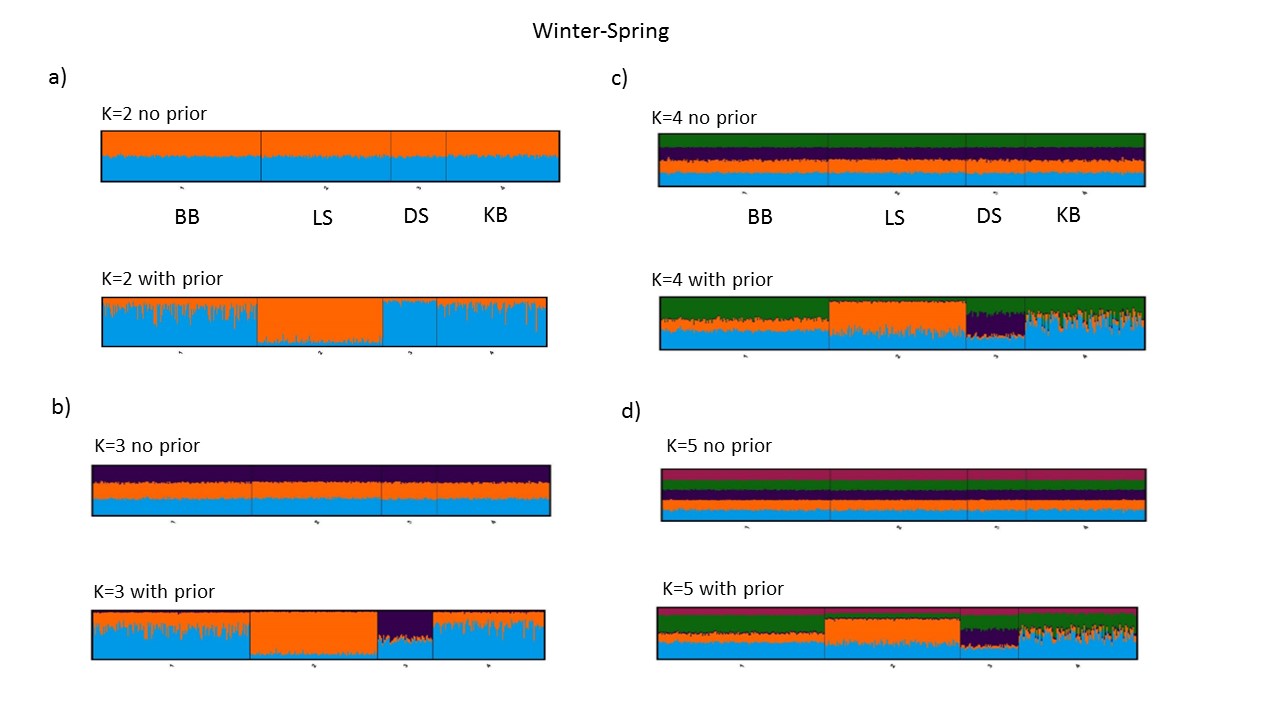


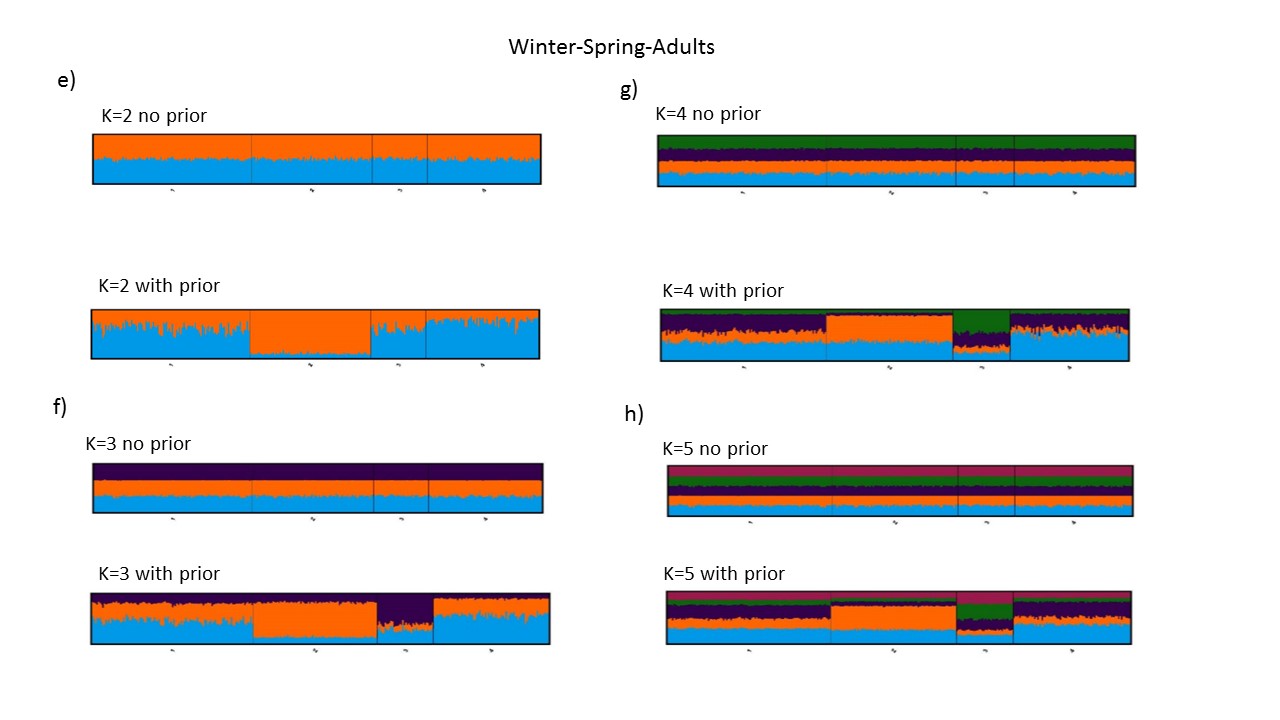


**Figure S4.** GENELAND heat maps showing the posterior probabilities of belonging to one of the identified groups for the Winter-Spring (all bears) and Winter-Spring Adults datasets (see Table 1). High posterior probability of the membership to a given cluster is indicated by lighter colors. Black dots indicate the GPS-locations of the individual sampling sites. Analyses used a contemporary sub-sample of genetic data from 402 biopsied, physically-captured, and harvested polar bears sampled during the winter and spring (Nov-May 2009-2014) representing polar bears from four neighboring subpopulations – BB, KB, LS and DS. We used samples collected during these two seasons as bears are widely distributed within their purported management boundaries and it excludes displacement of large groups of polar bears during the ice-free season. We assessed all bears in the Winter-Spring as well as Adults only, the latter being a sub-set of the Winter-Spring sample.


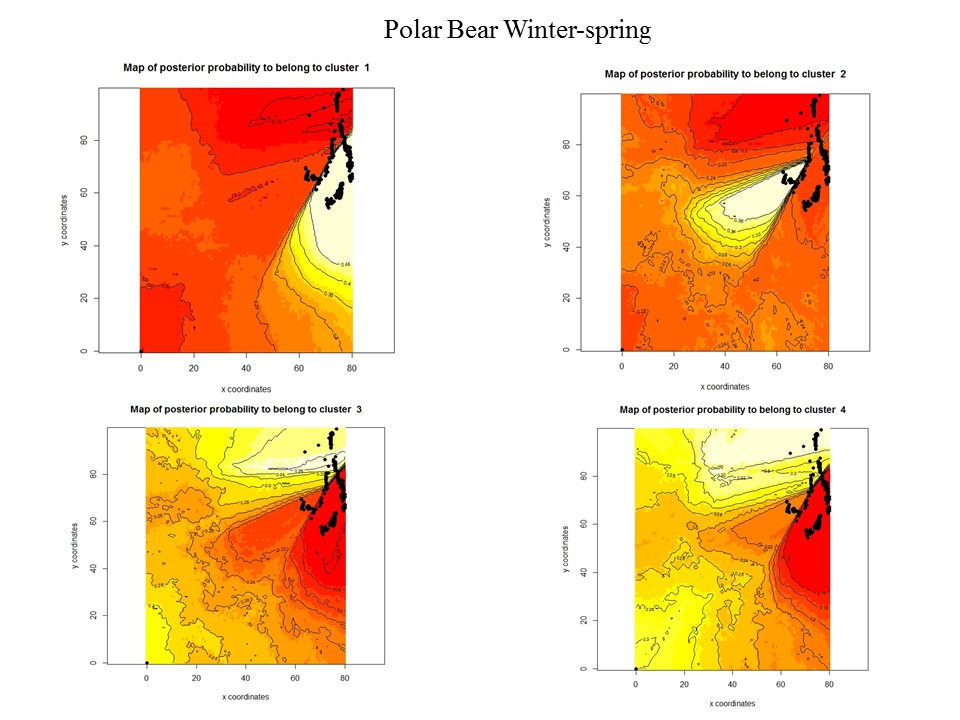


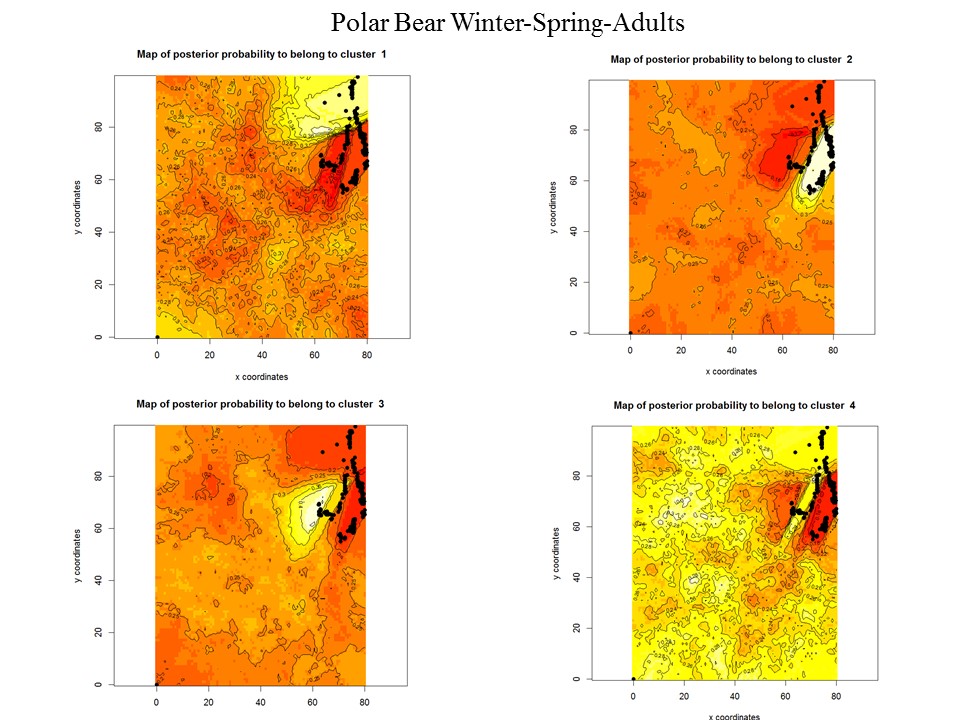


**Table S2.** Results of GENELAND analyses searching for groups using genetic relationship- linkage equilibrium and HWE- and geographical coordinates separating into a) Winter-Spring (all bears) and b) Winter-Spring Adults (a sub-set of the Winter-Spring sample).

| All bears | |  |  |  |
| --- | --- | --- | --- | --- |
|  | BB | LS | DS | KB |
| Group 1 | **91** | 0 | 0 | **96** |
| Group 2 | 3 | 1 | **44** | 0 |
| Group 3 | 35 | **113** | 5 | 3 |
| Group 4 | **11** | 0 | 0 | 0 |
|  |  |  |  |  |
| All Adults | | |  |  |
|  | BB | LS | DS | KB |
| Group 1 | 10 | **84** | 3 | 1 |
| Group 2 | **65** | 0 | 0 | **77** |
| Group 3 | **27** | 0 | **35** | 0 |
| Group 4 | 7 | 0 | 0 | 0 |

**References**

Chen C, Durand E, Forbes F, Franςois O. 2007. Bayesian clustering algorithms ascertaining spatial population structure: a new computer program and a comparison study. Molecular Ecology Notes 7:747–756.

Earl DA, VonHoldt BM. 2012. STRUCTURE HARVESTER: a website and program for visualizing STRUCTURE output and implementing the Evanno method. Conservation Genetic

Resources 4: 359–361

Evanno G, Regnaut S, Goudet J. 2005. Detecting the numberof clusters of individuals using the software structure: a simulation study. Molecular Ecology 14: 2611–2620

Goudet J. 1995. FSTAT 2.9.3.1: a computer program to calculate F statistics. Journal of Heredity 86: 485-486

Guillot G, Mortier F, Estoup A 2005. Geneland: a computer package for landscape genetics. Molecular Ecology Notes 5: 712-715.

Guillot 2008. Inference of structure in subdivided populations at low levels of genetic differentiation. The correlated allele frequencies model revisited. Bioinformatics 24: 2222-2228.

Guillot G, Santos F. 2009. A computer program to simulate multilocus genotype data with spatially auto-correlated allele frequencies. Molecular Ecology Resources 9(4): 1112-1120.

Jombart T. 2008. Adegenet: a R package for the multivariate analysis of genetic markers. Bioinformatics 24: 1403-1405.

Jombart T, Devillard S, Balloux F. 2010. Discriminant analysis of principal components: a new method for the analysis of genetically structured populations. BMC Genetics 11:94.

Kopelman NM, Mayzel J, Jakobsson M, Rosenberg NA, Mayrose I. 2015. Clumpak: a program for identifying clustering modes and packaging population structure inferences across K. Molecular Ecology Resources 15: 1179–1191.

Latch EK, Dharmarajan G, Glaubitz JC, Rhodes Jr. OE. 2006. Relative performance of Bayesian clustering software for inferring population substructure and individual assignment at low levels of population differentiation. Conservation Genetics 7: 295–302.

Paetkau, D. 2003. An empirical exploration of data quality in DNA-based population inventories. Molecular Ecology 12: 1375–1387.

Paetkau D, Amstrup SC, Born EW, Calvert W, Derocher AE, Garner GW, Messier F, Stirling I, Taylor MK, Wiig Ø, Strobeck C. 1999. Genetic structure of the world’s polar bear populations. Molecular Ecology 8:1571–84.

Peakall R, Smouse PE. 2006. GENALEX 6: genetic analysis in Excel. Population genetic software for teaching and research. Molecular Ecology Notes 6: 288-295.

Peakall R, Smouse PE. 2012. GenAlEx 6.5: genetic analysis in Excel. Population genetic software for teaching and research-an update. Bioinformatics 28, 2537-2539.

Pritchard JK, Stephens M, Donnelly P. 2000. Inference of population structure using multilocus genotype data. Genetics 155: 945-959.

Rambaut A, Suchard MA, Xie D & Drummond AJ 2014. Tracer v1.6, Available from http://beast.bio.ed.ac.uk/Trac.

Rice WR. 1989. Analyzing tables of statistical tests. Evolution 43: 223-225.

Wang J. 2017. The computer program STRUCTURE for assigning individuals to populations: easy to use but easier to misuse. Molecular Ecology Resources 17: 981-990.
